# The Macrophage Activator GcMAF-RF Enhances the Antitumor Effect of Karanahan Technology through Induction of M2–M1 Macrophage Reprogramming

**DOI:** 10.1155/2024/7484490

**Published:** 2024-02-29

**Authors:** Vera S. Ruzanova, Svetlana S. Kirikovich, Evgeniy V. Levites, Anastasia S. Proskurina, Evgeniya V. Dolgova, Genrikh S. Ritter, Yaroslav R. Efremov, Tatyana D. Dubatolova, Alexander V. Sysoev, Danil I. Koleno, Alexandr A. Ostanin, Elena R. Chernykh, Sergey S. Bogachev

**Affiliations:** ^1^Institute of Cytology and Genetics, Siberian Branch of the Russian Academy of Sciences, Novosibirsk, Russia; ^2^Novosibirsk National Research State University, Novosibirsk, Russia; ^3^Institute of Molecular and Cellular Biology, Siberian Branch of the Russian Academy of Sciences, Novosibirsk, Russia; ^4^N.N. Vorozhtsov Novosibirsk Institute of Organic Chemistry, Siberian Branch of the Russian Academy of Sciences, Novosibirsk, Russia; ^5^Research Institute of Fundamental and Clinical Immunology, Novosibirsk, Russia

## Abstract

Macrophages are the immune cells of high-immunological plasticity, which can exert both pro- and anti-inflammatory activity, as well as repolarize their phenotype to the opposite or neutral one. In this regard, M2 macrophages of the tumor-associated stroma (TAS) are a promising therapeutic target in treating malignant neoplasms. Using FACS assay, we have estimated the CD11b+/Ly-6G+/Ly-6C+ fraction of macrophages from the peritoneum and TAS in intact healthy mice and those with developed Lewis carcinoma, both untreated and treated according to Karanahan technology in combination with group-specific macrophage activator (GcMAF-RF). As well, the pattern of pro- and anti-inflammatory cytokines mRNA expression in different groups of experimental and tumor-bearing animals was assessed. It was found that: (i) exposure of intact mice to GcMAF-RF results in the increased number of CD11b+/Ly-6C+ peritoneal macrophages and, at the same time, the expression pattern of cytokines in peritoneal macrophages switches from that characteristic of the mixed M1/M2 phenotype to that characteristic of the neutral M0 one; (ii) combination of Karanahan technology and GcMAF-RF treatment results in M0/M1 repolarization of TAS macrophages; (iii) in tumor-bearing mice, the response of peritoneal macrophages to such a treatment is associated with the induction of anti-inflammatory reaction, which is opposite to that in TAS macrophages.

## 1. Introduction

Studies [1, 2] have characterized a new antitumor potential of Karanahan technology—the ability to induce an antitumor immune response. Karanahan technology is described in detail in [2]; it is time-ordered treatment of tumor-bearing mice with a cross-linking cytostatic cyclophosphamide and a complex double-stranded DNA agent. This treatment eradicates tumor stem cells from the tumor growth site, induces large-scale apoptosis of committed tumor cells, and activates antitumor immune responses.

Introduction of a the Gc protein-derived macrophage activating factor (GcMAF-RF) into the therapy was shown to synergistically enhance the antitumor effect of Karanahan technology. Based on the functional properties of GcMAF-RF, its antitumor effect may be associated with the activation of immune responses by inhibiting the immunosuppressive capabilities of myeloid cells, such as tumor-associated macrophages and myeloid-derived suppressor cells (MDSCs), in the tumor microenvironment; the most important element of the synergistic contribution of GcMAF-RF to tumor lysis is related to its capability of inhibiting the synthesis of protumor factors suppressing tumor-reactive iimmune responses in the tumor and its stroma. At this point, M2 macrophages become the target of GcMAF-RF, and repolarization of the M2 protumor phenotype to the M1 antitumor or M0 not-activated phenotype occurs [1].

The tumor stroma contains two types of cells exhibiting protumor properties: T regulatory cells and tumor-associated macrophages.

Chronometric/metronomic low-dose cyclophosphamide chemotherapy is known to differentially affect T regulatory CD25+/FOXP3+ cells and other T cell populations, completely eradicating suppressors from the tumor. This effect is believed to be associated with disturbances in the repair machinery in T regulatory cells, which prevents repair of chromatin breaks following cytostatic-induced interstrand crosslinks [3, 4]. In this case, T effector cells are preserved in sufficient quantities for an immune response to develop.

At the same time, chronometric/metronomic low-dose cyclophosphamide chemotherapy promotes the reversal of myeloid cells activity from tumor-supporting to tumor-reactive one, which is primarily controlled by switching the polarization of tumor-infiltrating macrophages from the M2 to M1 phenotype. The tumor-reactive properties of myeloid cells are significantly enhanced by the combination of chronometric/metronomic low-dose cyclophosphamide chemotherapy with CpG oligodeoxynucleotides (CpG ODN), anticancer vaccines, or cytokines [5–8]. Chronometric/metronomic low-dose cyclophosphamide chemotherapy alone or in combination with CpG immunomodulators, vaccines, or cytokines exerts an antitumor effect through the following mechanisms:

1. First of all, being a cytostatic, cyclophosphamide directly destroys tumor cells, inducing aberrant apoptosis;
2. In addition to direct cytotoxicity, chronometric/metronomic low-dose cyclophosphamide chemotherapy alone or in combination with immunomodulators of various classes activates the system of innate and adaptive immunity cells, which stimulates tumor cell elimination and tumor eradication;
3. Peripheral immune cells (neutrophils) are recruited to the tumor site;
4. Suppressor T regulatory cells are eliminated from the tumor site;
5. Myeloid cells switch their protumor activity to the tumor-reactive one; first of all, macrophages are repolarized from the M2 to M1 phenotype.

This diverse anticancer activity makes metronomic low-dose cyclophosphamide chemotherapy in combination with various immunomodulators a promising basic approach for the treatment of oncological diseases.

Our review [9] provides a conceptual diagram that indicates that chronometric/metronomic low-dose cyclophosphamide chemotherapy is a component of Karanahan cancer treatment technology. Karanahan technology is based on the main principles of chronometric/metronomic low-dose cyclophosphamide chemotherapy: a nontoxic dose of cyclophosphamide, timing of cytostatic administration, and a dendritic cell activator (a double-stranded DNA-based agent). This means that all antitumor effects of chronometric/metronomic low-dose cyclophosphamide chemotherapy, such as repolarization of protumor macrophages and neutrophils to a tumor reactive or neutral phenotype (M1(0)/N1(0) repolarization) and recruitment of peripheral immune cells, in particular neutrophils, to the tumor site, are fully implemented in Karanahan technology. In addition, large-scale apoptosis of committed tumor cells is induced, and tumor stem cells are eradicated from the tumor.

The purpose of this study was to investigate the phenotype of myeloid cells and the synthesis of mRNAs of basic pro- and anti-inflammatory cytokines and factors in the tumor microenvironment of mice receiving antitumor therapy using Karanahan technology alone and in combination with GcMAF-RF compared with the control tumor-bearing mice.

## 2. Materials and Methods

### 2.1. Experimental Animals

We used male and female 2- to 6-month-old C57BL/6 mice (weight, 18–24 g) bred at the Common Use Center Vivarium for Conventional Animals of the Institute of Cytology and Genetics of the Siberian Branch of the Russian Academy of Sciences (Novosibirsk, Russia). The animals were kept in groups of 6–10 mice per cage with free access to food and water. The animals were sacrificed using the method of cervical dislocation. All animal experiments were approved by the Animal Care and Use Committee of the Institute of Cytology and Genetics SB RAS (Protocol N 8 from 19 March 2019) and conducted in compliance with the national and international guidelines for the care and humane handling of laboratory animals.

### 2.2. Tumor Model

Lewis carcinoma is a weakly immunogenic tumor with high-metastatic activity [10]. Intramuscular engraftment of 2 × 10^6^ cells into the femoral region of C57BL/6 mice was performed. Lewis lung carcinoma occurred spontaneously in C57BL/6 mice in 1951. The strain was received from the National Cancer Institute of the USA in September, 1973. The tumor consists of polymorphic cells, most of which have a round shape.

### 2.3. Composite Double-Stranded DNA

The composite double-stranded DNA preparation (DNAmix) is a mixture of native and modified genomic DNA isolated from human placenta and salmon sperm and fragmented to a size of 200–6,000 bp. DNAmix is the subject of industrial properties of “KARANAHAN” LLC.

### 2.4. GcMAF-RF

The protocol for obtaining group-specific macrophage activator (GcMAF-RF) is described in detail in [1, 11]. GcMAF-RF is the subject of industrial property of “ACTIVATOR MAF” LLC.

### 2.5. Treatment of Lewis Carcinoma

The experiments were carried out in Novosibirsk (55°02′29″ N, 82°56′04″ E). A total of 2 × 10^6^ Lewis carcinoma cells were grafted intramuscularly into the right femoral region of mice. Three groups of mice were formed: control group (tumor-bearing mice); Karanahan Group (animals that received cyclophosphamide and DNAmix injections); and Karanahan + GcMAF-RF Group (animals that received Karanahan therapy with additional administration of GcMAF-RF). When the tumor reached 216–512 mm^3^ in size, mice were injected with the agents. The Karanahan Group received 100 mg/kg of cyclophosphamide (CP) intraperitoneally at 0, 28, 56, and 96 hr and 0.5 mg/mouse of DNAmix in 200 *µ*L of saline intratumorally at 18, 46, 74, and 114 hr. The Karanahan + GcMAF-RF Group received the same treatment with additional intratumoral injections of 2 *µ*g GcMAF-RF three hrs prior to each CP administration; after the final treatment with DNA preparation, GcMAF-RF was injected thrice a day for 3 days (115, 119, 124, 138, 143, 148, 162, 167, and 172 hr) and once a day for the remaining 2 days (191 and 215 hr). Tumor-bearing mice received similar injections of saline. The procedure used to prepare GcMAF-RF was thoroughly described in [1].

### 2.6. Isolation of Tumor Cells and Peritoneal Macrophages

When isolating Lewis carcinoma cells, the tumor was comminuted; the cells were resuspended in phosphate-buffered saline (PBS; Medigen, Novosibirsk, Russia), filtered through 40 *µ*m Nylon Cell Strainer (Falcon, Corning, USA), and their quantity was counted using a Goryaev's chamber. Peritoneal macrophages were washed out using a PBS from the peritoneal cavity and counted in the Goryaev's chamber.

### 2.7. Preparation of Tumor Smears

The tumor was surgically resected from mice; a tumor section was imprinted on the glass slide several times. Tumor smears were fixed with methanol during 7–10 min; staining was performed using Giemsa reagent (Sigma) at 1 : 10 dilution with deionized water during 30 min. An analysis was performed on a Leica DM 400B microscope.

### 2.8. Activation of Peritoneal Macrophages with GcMAF-RF and Lipopolysaccharide (LPS) Inducers

Peritoneal macrophages were isolated from intact mice and precipitated by centrifugation at 400 *g* for 7 min. The cells were resuspended in RPMI; their quantity was counted using a Goryaev's chamber. The cells (2 × 10^6^) were incubated in 1 mL of RPMI (intact peritoneal macrophages), 1 mL of RPMI supplemented with 2.5 *µ*g of GcMAF-RF (GcMAF-RF-activated peritoneal macrophages), and 1 mL of RPMI supplemented with 5 *µ*g of LPS (LPS-activated peritoneal macrophages) during 3 hr.

### 2.9. An Analysis of Changes in MDSC Population Size in the Tumor and among Peritoneal Macrophages

To assess changes in MDSC population size after Karanahan therapy and Karanahan therapy in combination with GcMAF-RF, the tumor was isolated on Days 15 and 22 since the treatment initiation, and peritoneal macrophages were isolated on Day 16. To block nonspecific binding, PBS containing 10% fetal bovine serum was added to all cells and incubated for 10 min at room temperature. The studied cells (2 × 10^5^) were incubated with 0,25 *µ*g of antibodies and isotype controls at room temperature in the dark for 30–60 min. The following antibodies were used (BioLegend, San Diego, CA, USA, cat. No. is presented in parentheses): suppressor cells of myeloid origin—APC anti-mouse/human CD11b Antibody (101212), PE anti-mouse Ly-6G Antibody (127607), and FITC anti-mouse Ly-6C Antibody (128005). The following isotype controls were used (BioLegend, cat. No is presented in parentheses): APC Rat IgG2b, *κ* Isotype Ctrl (400612), PE Rat IgG2a, *κ* Isotype Ctrl (400508), FITC Rat IgG2c, *κ* Isotype Ctrl (400705). Small tumor portions and some peritoneal macrophages were lyzed in TRIzol Reagent (Thermo Fisher Scientific, Waltham, USA) and stored at −70°C to further isolate RNA.

Flow cytometry analysis was carried out on a BD FACSAria III (Becton, Dickinson and Company, Franklin Lakes, USA) cell sorter at the Center for Collective Use of Flow Cytofluorometry of the Institute of Cytology and Genetics of the Siberian Branch of the Russian Academy of Sciences.

### 2.10. Isolation of Total RNA

We used tumor material and peritoneal macrophages obtained from mice to analyze changes in the number of immune cell populations in the experiments described in our early articles [1, 2]. Part of the tumor tissue and suspension of peritoneal macrophages were lysed and homogenized in TRIzol Reagent (Thermo Fisher Scientific, Waltham, USA) and stored at −20°C for 6–9 months. Total RNA from tumors and peritoneal macrophages was isolated following the manufacturer's instructions. The amount of RNA was measured on a Qubit 4 fluorometer (Thermo Fisher Scientific, Waltham, USA).

### 2.11. Obtaining cDNA

The reverse transcription PCR was carried out on a poly-A mRNA template using a T100 Thermal Cycler amplifier (Bio-Rad Laboratories, Inc., Hercules, USA) and an MMLV RT kit (Evrogen, Moscow, Russia) according to the manufacturer's protocol.

### 2.12. PCR Analysis

PCR primers for coding regions of each pro-inflammatory and anti-inflammatory cytokine gene were constructed using the Vector NTI v.9 software (Life Technologies, Wilmington, DE, USA) and synthesized by BIOSSET Ltd. (BIOSSET, Novosibirsk, Russia). The sequences of primers used in this study are listed in Table 1.

Polymerase chain reaction was carried out in a total volume of 50 *µ*L; the reaction mixture contained Taq-buffer, 2 mM MgCl_2_, 0.2 mM dNTP, 0.2 pmol of forward and reverse primers, 1 ng of the template, and 5 units of Taq-polymerase (the reagents for PCR were provided by Medigen, Novosibirsk, Russia).

The PCR scheme was as follows: cycle 1 (×1): 95°C – 3 min; cycle 2 (×33): 95°C – 30 s; 56°C (Arg1, Ido1, GAPDH)/59°C (IL-1*β*, TNF*α*, IL-12a, IL-10, TGF-*β*1, GAPDH)/64°C (NOS2, GAPDH) – 30 s; 72°C – 40 s; cycle 3 (×1): 72°C – 5 min; storage at 10°C.

The generated fragments were then detected by agarose gel electrophoresis.

### 2.13. Real-Time PCR

Real-time PCR was carried out in 96-well plates using BioMaster HS-qPCR SYBR (2х) (BIOLABMIX LLC, Novosibirsk, Russia) according to the manufacturer's protocol on a QuantStudio5 PCR system (Thermo Fisher Scientific, Waltham, USA). Real-time qPCR analysis of each sample was performed in three replicates. Individual results are provided for each mouse. The relative expression level was determined using the 2^–*ΔΔ*Сt^ method. Tumor-bearing mice not subjected to any treatment (or intact peritoneal macrophages) were used as the control group; the expression level of the target gene in them was assumed to be equal to 1. The GAPDH gene was used as reference. The cycling parameters were as follows: 95°C for 10 min, 40 cycles of 95°C for 30 s, 56°C (Arg1, Ido1, GAPDH)/59°C (IL-1*β*, TNF*α*, IL-12a, IL-10, TGF-*β*1, GAPDH)/64°C (NOS2, GAPDH) for 30 s, 72°C for 30 s, with a final melting step with slow heating from 6 to 95°C.

### 2.14. Statistical Analysis

Statistical analysis was performed using the Statistica 8 software (StatSoft, Tulsa, USA). The validity of differences was evaluated using the Mann–Whitney *U* test or analysis of four-field contingency tables. The revealed differences were considered statistically significant at *p* < 0.05 (Mann–Whitney *U* test) or *χ*^2^Pv  < 0.01 (analysis of four-field contingency tables). Statistical evaluation of real-time PCR analysis was carried out based on experimental and instrumental repetitions.

## 3. Results

### 3.1. Note to the Experimental Design

The main question to be answered was what happened to myeloid cells in the tumor microenvironment after application of Karanahan technology both alone and in combination with group-specific macrophage activator (GcMAF-RF) within the period of tumor resorption (15–16 days after therapy onset) in studies [1, 2]. Namely, whether M2 macrophages underwent repolarization toward tumor-neutral or tumor-reactive (M0/M1) states and tumor resorption was due to the activity of phagocytes located within the tumor node along the specified time span, or whether extravascular macrophages (e.g., peritoneal macrophages) or peripheral bloodstream neutrophils were recruited to the tumor site.

In this regard, we have assessed the functional phenotypes of macrophages and the mRNA expression of main diversely acting cytokines in tumor-associated stroma (TAS) cells of treated and untreated tumor-bearing mice, as well as in peritoneal macrophages of the same mice compared with intact healthy ones.

To scale and interpret the results obtained, we first assessed the phenotype and pattern of mRNA expression of cytokines and related to inflammation specific factors in peritoneal macrophages (which in concert with resident macrophages compose the extravascular immune system) of intact C57BL/6 mice in response to GcMAF, as well as to LPS, a standard macrophage activator. The analysis of peritoneal macrophages revealed a direct effect on the synthesis of specific mRNAs and a relationship between changes in the pull of synthesized mRNAs and the CD11b+Ly-6C+ phenotype.

Next, we evaluated the spectrum of cytokines and specific factors, which characterizes the protumor and antitumor phenotypes of tumor-infiltrating cells in the three analyzed mouse groups: untreated tumor-bearing mice, treated with Karanahan technology alone, and treated with Karanahan technology in combination with GcMAF-RF. The evaluation was performed on Days 15 or 16 after the onset of therapy, in the phase of explicit tumor resorption.

The comparison of synthesized mRNAs in TAS cells and peritoneal macrophages allowed to estimate the repolarizing effect of GcMAF-RF on the functional phenotype of macrophages inside and outside the tumor microenvironment compartment. The CD11b+Ly-6C+ phenotype is known to characterize both tumor-reactive M1 and protumor M2 macrophages. These populations are distinguished based on either sedimentation characteristics or production of specific factors with cytolytic or suppressor properties [12].

In an independent experiment, the phenotype and spectrum of synthesized mRNAs of cytokines and specific factors of peritoneal macrophages (also in three groups), as an extravascular cell population, were assessed on Day 16 after the start of therapy.

The evaluation revealed that, according to the spectrum of synthesized mRNAs of the analyzed factors, CD11b+Ly-6C+ resorbing tumor cells were not related to peritoneal macrophages and probably were local tumor-associated stromal macrophages. This means that altered TAS macrophages, but not recruited peritoneal macrophages, are involved in tumor resorption. Abundant purulence in the resorbing tumor, found in two of the four experiments, also suggests the involvement of recruited neutrophils in tumor destruction. The observed differences in neutrophil levels in the resorbing tumor are supposed to be associated with seasonal or annual cycles.

Experimental studies indicate that GcMAF-RF induces changes in macrophages, but not in neutrophils, and all changes revealed by the analysis of marker modalities are associated with the effect on CD11b+Ly-6C+ macrophages, both TAS and peritoneal macrophages. Nevertheless, the paper also provides data on the evaluation of changes in the level of cells in the CD11b+Ly-6G+ granulocytic neutrophil population.

### 3.2. Retrospective Comparative Analysis of Populations of CD11b+Ly-6G+/Ly-6C+ Tumor-Infiltrating Myeloid Cells in Several Successive Experiments (Treatment of Mice with Experimental Lewis Carcinoma Using Karanahan Technology Combined with GcMAF-RF)

**Figure 1:**
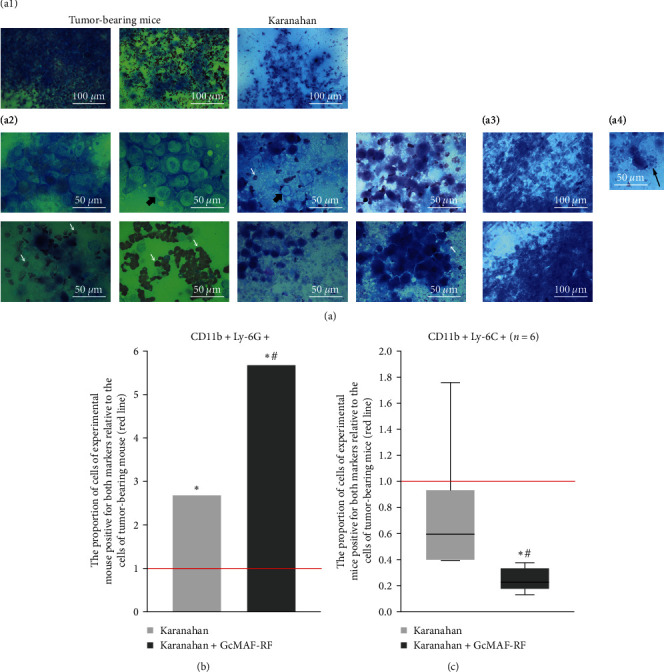
Analysis of immune cell populations isolated from tumors of tumor-bearing mice and mice treated with Karanahan technology solely and in combination with group-specific macrophage activator (GcMAF-RF). (a) Cytological analysis of tumor smears. Tumor smears were prepared from residual detectable tumors selected isolated/obtained from groups of tumor-bearing mice and mice treated with Karanahan technology: (control group) the smear displays tumor cell clusters and occasional erythrocyte clusters, no other cellular elements were found; (experimental group) there are small amounts of viable tumor cells, a large number of erythrocytes and neutrophils are present in the field of view, a lot of mucus is seen on the smear. (a1) Scale bar = 100 *µ*m; (a2) scale bar = 50 *µ*m, tumor cells are pointed by the thick black arrow, red blood cells are pointed by the white arrow; (a3) –neutrophil clusters, scale bar = 100 *µ*m; (a4) a neutrophil is pointed by the thin black arrow, scale bar = 50 *µ*m. In the group of synergistic treatment with Karanahan technology in combination with GcMAF-RF, there is depletion of tumor cells and the presence of erythrocytes, other cellular elements of the blood are not detected. (b) Evaluation of the immune response induced by treatment with Karanahan technology and Karanahan + GcMAF-RF in mice with Lewis carcinoma. Content of CD11b+Ly-6G+ cells in tumors isolated from mice of experimental groups on day 15 after the beginning of the therapy relative to that in tumors isolated from untreated tumor-bearing mouse (indicated by a the red line). The black asterisk denotes the difference with control (tumor-bearing mouse); the hash denotes the difference between experimental Karanahan and Karanahan + GcMAF-RF groups. For all comparisons, the level of significance is *χ*^2^Pv ≤ 0.01. (c) Evaluation of the immune response (content of cell populations (CD11b+Ly-6C+) induced by the treatment with Karanahan and Karanahan + GcMAF-RF technologies in mice with Lewis carcinoma (statistically significant sample of experimental animals). The median content of cell populations (CD11b+Ly-6C+) in tumors isolated from mice of experimental groups on day 15 or 16 after the beginning of therapy relative to tumor-bearing mice (indicated by a the red line). Box and whisker plot; the black asterisk denotes the significant difference with control (tumor-bearing mice); the hash indicates the significant difference between experimental Karanahan and Karanahan + GcMAF-RF groups, Pv  < 0.05; Mann–Whitney *U* test. The assessment was carried out based on the results of two independent experiments.

To analyze the events occurring in the tumor of experimental animals treated with Karanahan technology solely and in combination with GcMAF-RF [11], the results of four experimental series reported in [1, 2] were used: May 2020 (No. 1), November 2020 (No. 2), February 2021 (No. 3), and May 2021 (No. 4). We performed retrospective comparative analysis of changes in the population of myeloid cells carrying CD11b+Ly-6C+/Ly-6G+ markers. Changes detected on Days 15 or 16 after the start of the experiments were analyzed (Figure 1). It was related to the following. In the experiment no. 1, there was active purulence with massive pus release upon squeezing in mice of the Karanahan and Karanahan + GcMAF-RF groups starting from Day 15 after the onset of treatment. In the Karanahan + GcMAF-RF group, this phenomenon was much more pronounced and occurred in more mice.

In the experiment no. 2, no pronounced purulent discharge was observed; however, a large number of leukocytes were present in the tumor (Figures 1(a) and 1(b)). In the experiment no. 1, immune cell populations were not analyzed. The tumor site condition and animal survival time were assessed. The analysis of mouse survival revealed that Karanahan + GcMAF-RF therapy was significantly more effective than Karanahan therapy. The percentage of animals completely recovered from incurable Lewis carcinoma was 43% in the first case and 29% in the second case [1].

In the experiment no. 2, only the changes in immune cell populations (primary immunogram) in the tumor, blood, and spleen were evaluated. Flow cytometry analysis of tumor cells using side light scattering revealed two distinct major populations of monocytic and granulocytic cells [2]. Both populations carried markers characteristic of macrophages and neutrophils CD11b+Ly-6C+/Ly-6G+. The presence of CD11b+Ly-6G+ in the monocytic fraction was explained by an immature phenotype of myeloid neutrophil progenitors that lacked lytic granules. The presence of CD11b+Ly-6C+ in the granulocytic fraction detected by side light scattering might be explained by the identification of terminally differentiated macrophages or dendritic cells containing granules, which also express CD11b+Ly-6C+ surface markers on the cytoplasmic membrane. Phenotypic analysis showed an abrupt increase in the level of CD11b+Ly-6G+ granulocytes in the granulocytic fraction of the tumor, up to 80% (about 20% in the tumor-bearing mice Figure 1(b)). This fact may mean that peripheral phagocytes were recruited to the tumor site. In this case, the level of monocytes with CD11b+Ly-6C+ markers (macrophages) decreased.

In the first two experiments, an excess of leukocytes in the tumor site (Days 15–28), impeded the correct assessment of the state of tumor-associated CD11b+Ly-6G+/Ly-6C+ MDSCs not recruited (local) to the resorbing tumor due to the similarity of their superficial markers and those expressed by classically activated proinflammatory macrophages and neutrophils [12–14].

The following experiments nos. 3 and 4 demonstrated consistent data on changes in the level of CD11b+Ly-6G+/Ly-6C+ phagocytes (a significant decrease in CD11b+Ly-6C+), which indicated the lack of a pronounced saturation of the tumor site (recruitment to the tumor site) with peripheral cells bearing characteristic markers (Figure 1(c)).

The results of comparative analysis of the leukocyte influx into the tumor site and the state of cells with the CD11b+Ly-6G+/Ly-6C+ phenotype of the first two and second two identical experiments may mean a seasonal/annual cycle for TAS changes in such a way that a large-scale increase in the level of leukocytes in the tumor site was observed in the first two cases, whereas no similar effect occurred in the second two experiments. Importantly, elimination of the masking leukocyte recruitment due to natural environmental changes allows revealing the real changes in the MDSC population.

In the second experimental series, a significant decrease in CD11b+Ly-6C+ cells was clearly observed in the Karanahan + GcMAF-RF group. We suggest that if CD11b+Ly-6C+ tumor cells were replaced by recruited peritoneal macrophages of the same phenotype, the total number of cells would remain around the initial level.

Analysis of the plots the following events to be hypothesized:

1. A fraction of CD11b+Ly-6C+ cells might be removed from the tumor site, which indicates their high susceptibility to the treatment;
2. There was M2/M1, N2/N1 repolarization of phagocytes with loss of specific surface markers.

In both variants, this would be observed as a decrease (change) in the percentage of CD11b+Ly-6C+ cells.

As mentioned above, macrophages of any localization are the target for GcMAF-RF. In this regard, we interpret the observed mRNA expression pattern in the Karanahan + GcMAF-RF group exclusively in the context of the revealed changes in the CD11b+Ly-6C+ population. Changes in the CD11b+Ly-6G+ granulocytic neutrophil population might be secondary and related to the primary effect of GcMAF-RF on monocytic MDSCs.

The direction of these changes was determined by analyzing the mRNA synthesis of specific factors (cytokines, Arg1, NOS2, Ido1) in TAS cells of tumor-bearing and other groups of the experimental mice. The possibility of recruitment was assessed by the comparative analysis of synthesis of these mRNAs in TAS cells and extravascular phagocytes (peritoneal macrophages were analyzed).

In the absense of these cells, PCR-based estimation of the mRNA expression of investigated cytokines is expected to reveal a quantitative decrease in the content of MDSC-specific mRNAs in tumor specimens of treated tumor-bearing mice compared with those of untreated tumor-bearers.

Phenotype repolarization will be accompanied by multidirectional changes in the spectrum of synthesized mRNAs of pro- and anti-inflammatory TAS factors in the analyzed samples.

If the mRNA expression profile of the analyzed factors in TAS cells differs from that in peritoneal macrophages, a comparative analysis may allow identifying the type of cells in the tumor: either these are CD11b+Ly-6C+ myeloid cells of the tumor microenvironment, including tumor-associated macrophages, or these are CD11b+Ly-6C+ recruited macrophages. If the mRNA expression profile of the analyzed factors is the same both in TAS cells and in peritoneal macrophages, a comparative analysis will not allow to distinguish these two cell types from each other, making the detection of phagocytes recruitment into the tumor impossible.

To make all these comparisons, first of all, it was necessary to determine how the phenotype and spectrum of the synthesized peritoneal macrophage mRNAs of intact animals would change upon administering GcMAF-RF without additional treatment, i.e., to evaluate the net effect of GcMAF-RF.

### 3.3. Phenotype Characterization and PCR Analysis of the Synthesis of Specific Peritoneal Macrophage mRNAs Isolated from Mice, Intact and Treated with GcMAF-RF and LPS Inducers

The introduction of GcMAF-RF to the Karanahan treatment regimen in mice led to a significant increase in the efficacy of therapy. This factor is known to promote macrophages to phagocytic and increased lytic activity against tumor cells *in vitro* [1]. To assess the effect of GcMAF-RF on macrophages *ex vivo* (net effect), a series of experiments were performed to assess changes in the level of cells with CD11b+Ly-6G+/Ly-6C+ markers characteristic of MDSCs. For analysis, the C57BL/6 mouse line was used. A single compound dose of 2 *μ*g target protein was chosen.

**Figure 2:**
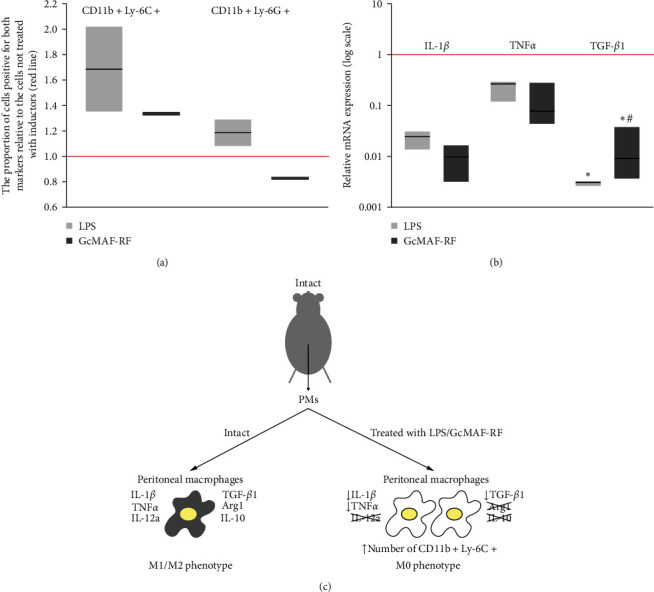
Characterization of the phenotype and synthesis of specific pro- and anti-inflammatory factor mRNAs in the peritoneal macrophages (PMs) isolated from mice, intact and activated with lipopolysaccharide (LPS) and GcMAF-RF. (a) The content of CD11b+Ly-6C+ and CD11b+Ly-6G+ cells (markers characteristic of myeloid suppressor cells) in populations of peritoneal macrophages isolated from intact mice and exposed to medium supplemented with either LPS or GcMAF-RF, relative to that in untreated cells (indicated by a red line). Floating bars (min to max); line at median. The evaluation experiment was performed with two mice in each group. (b) Relative levels of mRNA expression of some pro- and anti-inflammatory factor genes in peritoneal macrophages of intact animals activated with GcMAF-RF and LPS compared with peritoneal macrophages of intact animals not treated with activators (their expression level = 1; the Y-axis is logarithmic). The black asterisk denotes the significant difference from intact cells; the hash indicates the significant difference between cells activated with lipopolysaccharide (LPS) and GcMAF-RF inducers, Pv  < 0.05; Mann–Whitney *U* test. Floating bars (min to max); line at median; *n* = 5, pooled sample. (c) Graphical representation of the relative levels of mRNA expression of some pro- and anti-inflammatory factor genes in the population of peritoneal macrophages, isolated from an intact mouse, after their incubation in medium, medium containing lipopolysaccharide (LPS), or medium containing group-specific macrophage activator (GcMAF-RF). Evaluation of peritoneal macrophage phenotype changes after treatments. The figure schematically shows macrophages. An increase in the number of schematic cells indicates an increase in the number of CD11b+Ly-6C+ monocytic cells among peritoneal macrophages. gray color denotes the M1/M2 phenotype of cells releasing both pro- and anti-inflammatory cytokines; white color denotes the normal M0 phenotype. A factor (cytokine) name without an arrow means its presence in a sample. A crossed-out cytokine name means its absence in a sample. The presence/absence in a sample was assessed by polymerase chain reaction with specific primers. Relative levels of mRNA expression of pro- and anti-inflammatory cytokine genes in LPS/GcMAF-RF-treated peritoneal macrophages compared with those in intact peritoneal macrophages were assessed by Real-time PCR. A factor (cytokine) name with an up (down) arrow means an increase (decrease) in a relative level of cytokine mRNA expression compared with that in intact peritoneal macrophages.

Mouse peritoneal macrophages were found to express specific CD11b+Ly-6G+/Ly-6C+ marker molecules. Exposure of cells to GcMAF-RF and LPS (a common macrophage activator) resulted in an increase in the level of cells with the CD11b+ Ly-6C+ phenotype (Figure 2(a)). In this case, the level of GcMAF-RF-treated peritoneal cells with D11b+Ly-6G+ granulocyte markers decreased. For LPS, this marker feature changed indefinitely and multidirectionally. By their side light scattering properties, peritoneal macrophages look like a single population. The presence of two macrophage and granulocyte markers without distinctive morphological differences, which respond differently to treatment, may mean that the initial population is represented by immature cells of two phagocyte populations. Treatment with GcMAF-RF and LPS stimulates maturation of immature monocytes, which leads to an increase in CD11b+Ly-6C+ cells. The level of cells in the CD11b+Ly-6G+ population does not increase upon treatment with GcMAF-RF, which indicates the lack of an activating effect of GcMAF-RF on the granulocytic peritoneal macrophage fraction.

The conducted quantitative real-time-PCR-based assessment of TGF-*β*1, TNF*α*, and IL-1*β* mRNA expression and qualitative PCR-based one of Arg1, IL-12a, IL-10, Ido1, and NOS2 mRNA expression in peritoneal macrophages of untreated animals and those affected with GcMAF-RF and LPS indicate that: (i) peritoneal macrophages of untreated animals express mRNAs for L-1b, IL-12, TGF-*β*1, Arg1, TNF*α*, IL-10, Ido1, NOS2, i.e., factors facilitating opposite aspects of the inflammation (qualitative analysis, data not shown); (ii) both compounds exert the similar effect on mRNA expression of the tested factors.

Transcriptional activity of all analyzed genes active in peritoneal macrophages of intact animals is inhibited (Figures 2(b) and 2(c)). The activity of genes of PCR-analyzed cytokines indicates that LPS and GcMAF-RF “calm down” the synthetic, both suppressive and proinflammatory, activity of peritoneal macrophages.

Comparison of the level of CD11b+Ly-6C+ macrophages and synthesis of the analyzed cytokines suggests that treatment with GcMAF-RF at the selected experimental dose induces an increase in the level of phagocytes with the indicated marker, and the spectrum of synthesized peritoneal macrophage mRNAs changes from a mixed M1/M2 phenotype (untreated peritoneal macrophages) to a neutral M0 phenotype (Figure 2).

### 3.4. PCR Analysis of mRNA Synthesis by Cells in a Reducing Tumor on Day 15/16 of the Experiment

The main objective of this study was to evaluate changes in cells of a reducing tumor after treatment with Karanahan technology and in combination with GcMAF-RF.

This analysis was performed with cryopreserved mouse tumor samples from experiments 3 and 4 where there was no significant increase in the level of leukocytes (neutrophils) in TAS. This fact meant that the obtained results would be more relevant to local TAS cells, but not to recruited phagocytes.

**Figure 3:**
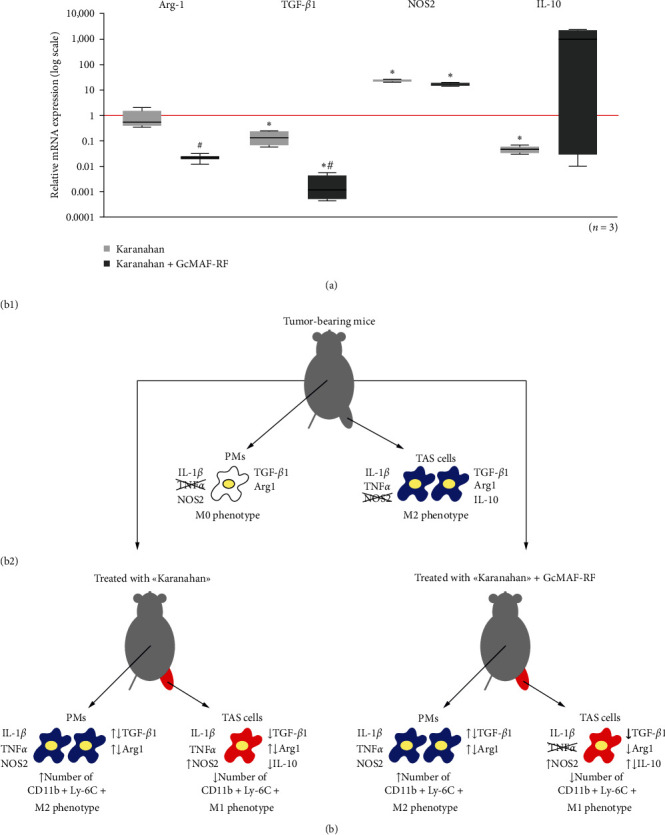
Analysis of synthesis of specific pro- and anti-inflammatory factor mRNAs in reducing tumors isolated from tumor-bearing mice and mice treated with Karanahan technology and in synergy with GcMAF-RF. (a) Relative levels of mRNA expression of some pro- and anti-inflammatory factor genes in populations of tumor cells isolated from mice treated with Karanahan technology and in synergy with GcMAF-RF compared with cells isolated from tumor-bearing mice (their expression level = 1; the Y-axis is logarithmic). Box and whisker plot; the black asterisk denotes the significant difference with control (tumor-bearing mice); the hash indicates the significant difference between experimental Karanahan and Karanahan + GcMAF-RF groups, Pv  < 0.05; Mann–Whitney *U* test. (b) Graphical representation of the relative levels of mRNA expression of some pro- and anti-inflammatory factor genes in peritoneal macrophages (PMs) and cells of tumor-associated stroma (TAS) isolated from tumor-bearing mice, mice treated with Karanahan technology, and mice treated with Karanahan technology in synergy with GcMAF-RF. (b1) The figure schematically shows peritoneal macrophages and TAS macrophages of tumor-bearing mice. Blue color denotes the M2 anti-inflammatory phenotype; white color denotes the normal M0 phenotype. A factor (cytokine) name without an arrow means its presence in a sample. A crossed-out cytokine name means its absence in a sample. The presence/absence in a sample was assessed by polymerase chain reaction with specific primers. (b2) Evaluation of peritoneal macrophages and TAS cells phenotype changes after treatments. The figure schematically shows peritoneal macrophages and TAS macrophages of mice treated with Karanahan technology alone and in synergy with GcMAF-RF. An increase/decrease in the number of schematic cells after treatments indicates an increase/decrease in the number of CD11b+Ly-6C+ monocytic cells among peritoneal macrophages and TAS cells. Blue color denotes the M2 anti-inflammatory phenotype; red color denotes the M1 proinflammatory phenotype. Relative levels of mRNA expression of pro- and anti-inflammatory factor (cytokine) genes in peritoneal macrophages and TAS cells of mice treated with Karanahan technology alone and in synergy with GcMAF-RF compared with those in peritoneal macrophages and TAS cells of tumor-bearing mice were assessed by Real-time PCR. A factor (cytokine) name with an up (down) arrow means an increase (decrease) in a relative level of factor (cytokine) mRNA expression in cells isolated from treated mice compared with that in cells isolated from tumor-bearing mice. A factor (cytokine) name with bidirectional arrows means multidirectional changes in a relative level of the factor mRNA in cells isolated from treated mice compared with that in cells isolated from tumor-bearing mice. The bold arrow indicates additive inhibition of TGF-*β*1 expression under the synergistic action of Karanahan technology and GcMAF-RF.

The qualitative PCR-based assessment of TGF-*β*1, Arg1, NOS2, IL-10, TNF*α*, IL-1*β*, IL-12a, and Ido1 mRNA expression in cells of the tumor node, conducted on Days 15 and 16 since the treatment initiation, indicated that the inclusion of GcMAF-RF into the treatment course results in suppressing the synthesis of anti-inflammatory cytokine mRNAs. At the same time, it does not abrogate the stimulating effect of Karanahan on the production of iNOS synthase mRNA, a hallmark of the proinflammatory capabilities of macrophages under the sustained expression of IL-1*β* mRNA (qualitative analysis, data not shown). The following factors: TGF-*β*1, Arg1, NOS2, IL-10, mRNAs of which were consistently detected in all standard PCR assays, were further assessed using real-time PCR (Figure 3).

The results of real-time PCR indicate that the therapy leads to the following results: Karanahan technology alone causes inhibition of TGF-*β*1 suppressor mRNA synthesis does not affect the expression of Arg1. Karanahan technology + GcMAF-RF causes inhibition of TGF-*β*1 and Arg1 suppressor mRNA synthesis. For the TGF-*β*1 gene, the inhibitory effect is additive. Karanahan technology in combination with GcMAF-RF has a multidirectional effect on the activation of IL-10 mRNA synthesis. Karanahan technology enhances expression of the NO synthase 2 gene, and addition of GcMAF-RF to therapy does not abolish the expression of this gene. This fact may indicate the induction of nitric oxide synthesis and a tumor-reactive cytoreductive effect, i.e., M1 repolarization of MDSCs (Figure 3(a)).

In general, these qualitative and quantitative PCR assays imply that changes in suppressor activity of TAS cells (repolarization), assessed by mRNA synthesis of main suppressors at a certain day, depend on the animal and the treatment (the chosen compound dose), and appear as individual differences in the trait severity. This means that the trait in the group develops unevenly over the selected period of time, and that, perhaps, the chosen compound dose induces a multidirectional response in the synthesis of selected cytokines, depending on the individual response of the body to the treatment. It is manifested both by a decrease in the percentage of cells with a characteristic phenotype (Figure 1(c)) and in general by a decrease in the expression of main suppressor genes TGF-*β*1 and Arg1, and does not abrogate the elevated NOS2 expression, which classifies the changes in TAS an M1 repolarization (Figure 3(b))

### 3.5. Phenotype and Cytokine mRNA Expression of Peritoneal Macrophages after Treatment of Tumor-Bearing Mice with Karanahan and Karanahan + GcMAF-RF

To assess chemoattraction of peritoneal macrophages to the tumor site, we analyzed the content of cells with the CD11b+Ly-6C+ phenotype in the peritoneal macrophage and tumor MDSC populations in experimental animals and changes in the spectrum of pro- and anti-inflammatory cytokine and factor mRNAs synthesized by them.

**Figure 4:**
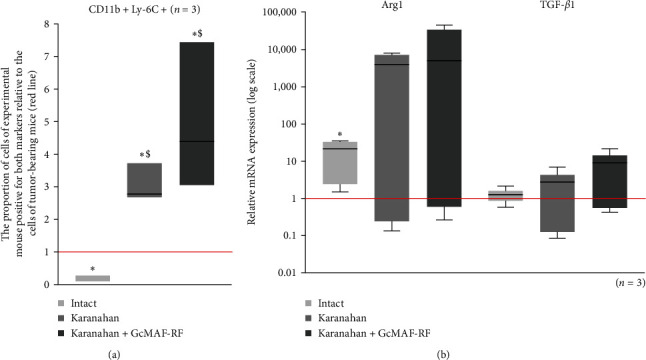
The phenotype and synthesis of specific anti-inflammatory factor mRNAs in the peritoneal macrophage population from intact mice, tumor-bearing mice, and tumor-bearing mice after treatment with Karanahan technology and in synergy with GcMAF-RF. (a) Characterization of the peritoneal macrophage phenotype isolated from intact mice, tumor-bearing mice, and mice treated with Karanahan technology and in synergy with GcMAF-RF. The results of flow cytometry analysis for myeloid-derived suppressor cell markers of monocytes (CD11b+Ly-6C+) of peritoneal macrophage populations isolated from intact mice and mice treated with Karanahan technology and in synergy with GcMAF-RF relative to tumor-bearing mice (indicated by a red line). Floating bars (min to max); line at median. The black asterisk denotes the significant difference with control (tumor-bearing mice); $—indicates the significant difference with intact mice, Pv  < 0.05; Mann–Whitney *U* test. (b) Relative levels of mRNA expression of some anti-inflammatory factor genes in peritoneal macrophage populations isolated from intact mice and mice treated with Karanahan technology and in synergy with GcMAF-RF in comparison with peritoneal macrophages isolated from tumor-bearing mice (their expression level = 1; the *Y*-axis is logarithmic). Box and whisker plot.

We repeated an extended version of the analysis originally performed in [1, 2]. Mice were treated with Karanahan and Karanahan + GcMAF-RF technologies. On Day 16 after the start of the experiment, mice were sacrificed, and peritoneal macrophages of all groups were isolated and analyzed (Figure 4). Both treatments were found to stimulate an increase in the level of cells with CD11b+Ly-6C+ markers in the peritoneal macrophage population (Figure 4(a)).

The qualitative PCR analysis indicates that both therapies activate the synthesis of mRNAs of both pro- and anti-inflammatory factors in peritoneal macrophages (qualitative analysis, data not shown). In this case, in three analyzed mice from a separate therapeutic group, PCR product formation is multidirectional and, as previously supposed, depends on the individual features of the animal in combination with the selected dose and the selected time of analysis after the treatment. In this regard, to characterize in detail the efficiency of mRNA expression, we chose two genes, Arg1 and TGF-*β*1, with distinctive differences in the efficiency of mRNA expression in intact mice, tumor-bearing mice, and treated experimental animals (Figure 4(b)). In addition, these are the most significant factors that mediate the immunosuppressive activity of TAS cells. Comparison of expression of these genes in peritoneal macrophages and TAS may provide an answer to the question of whether M2→M0/1 repolarization of TAS macrophages occurred, or whether vacant stromal niches were colonized by macrophages located outside the tumor site.

Despite a significant value dispersion, the results of real-time PCR assays revealed that the expression of Arg1 mRNA in peritoneal macrophages of mice of both groups was significantly higher than in tumor-bearing mice. The treatment conducted did not affect the expression of TGF-*β*1 mRNA, which retained at the same level both in healthy and in tumor-bearing animals. That is, the expression of Arg1 and TGF-*β*1 mRNA differ drastically in TAS cells (suppressed expression of both factors after GcMAF is being included into the treatment) and in peritoneal macrophages (increased arginase expression along with unaffected TGF-*β*1 mRNA synthesis). It means the treatments conducted facilitated the changes in TAS cells, which ensure a minimal feasibility of chemoattraction (infiltration) of peritoneal macrophages into TAS.

## 4. Discussion

As mentioned above, group-specific macrophage activator (GcMAF) induces changes in macrophages, but not in neutrophils, which is confirmed by the experimental studies. Our analysis of the effect of GcMAF-RF on the total peritoneal macrophage population also indicated that only cells with the CD11b+Ly-6C+ phenotype, but not CD11b+Ly-6G+ one, respond to induction by the activator. In this regard, we believe that all changes found in the analysis of marker modalities are associated with the effect on CD11b+Ly-6C+ macrophages of both phagocytes of TAS and peritoneal phagocytes.

M2 tumor-associated macrophages of various tumors account for 20%–40% of TAS myeloid cells. Tumor-associated macrophages play a key role in oncogenic processes, enhancing tumor cell proliferation, angiogenesis in the tumor site, and metastasis. They form a barrier to the cytotoxic effector function of T cells and natural killer cells [12, 15, 16]. During the tumor development phase, tumor-associated macrophages secrete anti-inflammatory factors, including IL-10, which stimulates polarization of naive T cells into T regulatory cells, and TGF-*β*1, which is the main cytokine that induces anergy of T cytotoxic and natural killer cells [17]. Tumor-associated macrophages form preniches for expansion by migrating tumor stem cells [16, 18, 19]. The presence of a large number of M2 macrophages in the tumor site is a negative prognostic sign. On the other hand, if M1 macrophages are present in the tumor, the prognosis is favorable [12, 16]. These facts indicate that myeloid cells with the M2 macrophage phenotype may be a successful target for anticancer therapy. The reductive effect on the M2 population will deprive the tumor of support by TAS-secreted factors and activate the tumoricidal potential.

The idea to affect TAS myeloid cells possessing immunosuppressive activity resulted in several principal approaches. These are complete elimination of these cells by relatively low doses of a cytostatic, induction of M2/M1 repolarization, blockade of activity of TAS cellular elements, and suppression of MDSC functional activity, including induction of maturation of TAS immature monocytes. TAS cells are inactivated by various compounds that affect the metabolic cascades of suppressors, which block various vital elements of their functioning [16, 20–23].

One of the promising ways to affect TAS M2 cells is to reprogram them to a tumor-reactive M1 or neutral M0 phenotype. Macrophages are functionally flexible cells that can alter their functional activity, depending on external conditions, due to changes in the metabolic processes [24]. There are several ways of metabolic reprograming/repolarization of TAS M2 macrophages, which include switching of fatty acid oxidation and glycolysis pathways to the aerobic pathway of energy production and alteration of the acidic pH of the tumor microenvironment through reducing the concentration of extracellular adenosine that, together with the agonistic adenosine receptor, plays a dominant role in adenosine-dependent differentiation of macrophages into the M2 phenotype [25, 26]. Repolarization of TAS M2 macrophages may involve TLR receptors and their agonists, proinflammatory cytokines, specific antibodies, RNAs, and small molecules [16].

The main and easily detectable criteria for M2–M1/0 macrophage repolarization are changes in mRNA expression or direct production of IL-10, TGF-*β*1, TNF*α*, and IL-1*β*. An additional criterion for M1 reprograming of M2 macrophages is believed to be an increase in their phagocytic index and iNOS synthesis.

In our previous studies, we discovered the third vector of Karanahan technology alone and in combination with the macrophage activator GcMAF-RF—activation of antitumor immunity [2, 11]. Analysis of a regressing tumor indicated an effect on TAS elements, namely dendritic cells, MDSCs, and T regulatory cells.

In this study, we evaluated the ability of applied mono and combination therapy to reprogram TAS M2 macrophages to the M1/0 phenotype with characteristic repolarization markers.

After a primary screening of synthesized cytokine and specific factor mRNAs, we focused on analysis of mRNA synthesis of IL-10, TGF-*β*1, and iNOS2 as factors that demonstrated an unambiguously interpreted response to the therapy and were important for the pro- or antitumor properties of TAS macrophages.

### 4.1. Peritoneal Macrophages of Intact Animals (GcMAF-RF Inducer)

We compared the changes in peritoneal macrophage surface markers after treatment with GcMAF-RF and LPS and the efficiency of mRNA synthesis of some pro- and anti-inflammatory factors. This comparison revealed a relationship between the CD11b+Ly-6C+/Ly-6G+ phenotype and the cytokine-producing activity of phagocytes after treatment with GcMAF-RF (peritoneal macrophages) without additional external influence (no body effect). Treatment with both activators (GcMAF-RF and LPS) at selected doses was found to lead to an increase in the level of cells with the CD11b+Ly-6C+ phenotype and a unidirectional decrease in mRNA synthesis of inflammatory mediators TNF*α*, IL-1*β*, and IL-12a and main suppressor factors TGF-*β*1, Arg1, and IL-10.

These results may be explained as follows: the population of peritoneal myeloid cells with the CD11b+Ly-6C+/Ly-6G+ phenotype comprises both mature macrophages and MDSCs. When exposed to selected activator doses, monocytic MDSCs immediately transform into macrophages with the M0 phenotype, giving an increase in cells with CD11b+Ly-6C+ surface markers, while mature macrophages present in the analyzed population with the same surface markers and possessing a multidirectional mixed (according to mRNA expression) M1/M2 phenotype are repolarized to M0 cells. This means M0 programing/reprograming of the active phagocytes.

The phagocytic index was determined for GcMAF-RF and LPS-activated peritoneal macrophages. Despite inhibition of total synthesis of pro- and anti-inflammatory factors, the cells were found to actively phagocytize metal beads, which is considered to be a feature of the M1 phenotype [1]. This indicates that the phagocytosis trait (M1) and the opposite direction of activator-induced cytokine-producing activity (M0) of peritoneal macrophages may either be combined in the same cell type or belong to functionally different cells of the peritoneal macrophage community. Probably, not all M1 cells were exposed to activators, which may also lead to mixing functionally multidirectional marker modalities.

### 4.2. Tumor-Associated Stroma (Karanahan and Karanahan + GcMAF-RF Treatment)

Analysis of treated tumor tissue indicates that TAS myeloid cells with protumor activity underwent M0/M1 repolarization. The macrophage activator is an effective inhibitor of TGF-*β*1 and Arg1 suppressor mRNA synthesis when it is used together with Karanahan technology. In this case, GcMAF-RF introduced into Karanahan therapy does not abolish high expression of the iNO synthase 2 gene, which indicates induction of nitric oxide synthesis and, upon decreasing arginase activity, the development of a tumor-reactive cytoreductive effect, i.e., M1 repolarization. Coexpression of two factors, NOX2, an enzyme catalyzing synthesis of free oxygen radicals (expressed predominantly by neutrophils) and iNOS2, is known to result in both ROS and NO. Nitric oxide peroxidation generates peroxynitrite that nitrosylates TCR, resulting in T cell anergy [12, 13, 27, 28]. Simultaneous deprivation of CD11b+Ly-6G+ neutrophils, main NOX2 producers, and activation of iNOS2, which is typical of macrophages, suggest decreasing oxygen free radicals and increasing nitric oxide in the tumor site [14]. NO synthesis by activated macrophages, which is not modified by excess ROS, is considered an important element of antitumor cytotoxicity, which indicates the M1 phenotype of phagocytes [27, 29, 30]. We suggest a pronounced M0/1 polarizing effect of GcMAF-RF.

Comparison of a decrease in the level of cells with the CD11b+Ly-6C+ phenotype in the tumor microenvironment in two experimental Karanahan and Karanahan + GcMAF-RF groups and an increase in CD11b+Ly-6C+ peritoneal macrophages in tumor-bearing animals after treatment with GcMAF-RF and the efficacy of synthesis of key immune cell suppressive factors (TGF-*β*1 and Arg1) indicates the lack of an intrinsic relationship between the CD11b+Ly-6C+ phenotype of cells and their cytokine/suppressor factor synthesizing activity. So, the CD11b+Ly-6C+ phenotype characterizes cells with different properties in TAS and peritoneal macrophages.

### 4.3. Peritoneal Macrophages of Tumor-Bearing Mice (Karanahan and Karanahan + GcMAF-RF Treatment)

A series of experiments to assess peritoneal macrophage chemoattraction to the tumor site revealed that both treatments stimulated an increase in the level of cells with CD11b+Ly-6C+ markers in the peritoneal macrophage population of treated animals from both groups, similar to the results of peritoneal macrophage activation by GcMAF-RF alone in intact animals. Herewith, the number of CD11b+Ly-6C+ cells in TAS of treated tumor-bearing mice is reliably reduced (Figure 1).

Moreover, data on the Arg1 and TGF-*β*1 (one of the main suppressors of immune cells) mRNA expression in peritoneal macrophages and in TAS cells of tumor-bearing mice, treated accordingly to Karanahan and Karanahan+GcMAF technologies, indicate that in peritoneal macrophages these treatments cause the elevated expression of arginase mRNA and have no effect on TGF-*β*1, while in TAS both factors are suppressed.

The collation of the facts above implies that the treatments resulted in some changes linked to the local TAS cells, with no connection with recruited peritoneal macrophages.

## 5. Conclusions

The study demonstrates the synergistic effect of tumor destruction due to the use of Karanahan technology in combination with the GcMAF-RF activator, which is associated with M2–M1 reprograming of tumor-associated macrophages.

## Figures and Tables

**Table 1:** The sequences of primers used in this study.

Primers	Oligonucleotide sequences
IL-1*β*-for	5'-TCCAGGATGAGGACATGAGCAC-3'
IL-1*β*-rev	5'-GAACGTCACACACCAGCAGGTTA-3'
TNFa-for	5'-AAGCCTGTAGCCCACGTCGTA-3'
TNFa-rev	5'-GGCACCACTAGTTGGTTGTCTTTG-3'
IL-10-for	5'-GACCAGCTGGACAACATACTGCTAA-3'
IL-10-rev	5'-GATAAGGCTTGGCAACCCAAGTAA-3'
IL-12a-for	5'-TGTCTTAGCCAGTCCCGAAACC-3'
IL-12a-rev	5'-TCTTCATGATCGATGTCTTCAGCAG-3'
TGF-b-1-for	5'-GTGTGGAGCAACATGTGGAACTCTA-3'
TGF-b-1-rev	5'-TTGGTTCAGCCACTGCCGTA-3'
NOS2-for	5'-TCACCTTCGAGGGCAGCCGA-3'
NOS2-rev	5'-TCCGTGGCAAAGCGAGCCAG-3'
Arg1-for	5'-GATTATCGGAGCGCCTTTCT-3'
Arg1-rev	5'-CCACACTGACTCTTCCATTCTT-3'
Ido1-for	5'-AGGATCCTTGAAGACCACCA-3'
Ido1-rev	5'-CCAATAGAGAGACGAGGAAG-3'
GAPDH-for	5'-AAATGGTGAAGGTCGGTGTG-3'
GAPDH-rev	5'-TGAAGGGGTCGTTGATGG-3'

for, a forward primer; rev, a reverse primer.

## Data Availability

The data used to support the findings of this study are included within the article.
